# Validation of the UCLA Child Post traumatic stress disorder-reaction index in Zambia

**DOI:** 10.1186/1752-4458-5-24

**Published:** 2011-09-24

**Authors:** Laura K Murray, Judith Bass, Elwyn Chomba, Mwiya Imasiku, Donald Thea, Katherine Semrau, Judith A Cohen, Carrie Lam, Paul Bolton

**Affiliations:** 1 Johns Hopkins University Bloomberg School of Public Health, Dept. of International Health, Baltimore, MD, USA; 2 Johns Hopkins University Bloomberg School of Public Health, Dept. of Mental Health, Baltimore, MD USA; 3 University Teaching Hospital, Lusaka, Zambia; 4 University of Zambia, Department of Psychology, Lusaka, Zambia; 5 Boston University School of Public Health, Boston, MA USA; 6 Allegheny General Hospital, Center for Traumatic Stress in Children & Adolescents, Pittsburgh PA, USA

**Keywords:** PTSD, assessment validation, children, low resource country, mental health

## Abstract

**Background:**

Sexual violence against children is a major global health and human rights problem. In order to address this issue there needs to be a better understanding of the issue and the consequences. One major challenge in accomplishing this goal has been a lack of validated child mental health assessments in low-resource countries where the prevalence of sexual violence is high. This paper presents results from a validation study of a trauma-focused mental health assessment tool - the UCLA Post-traumatic Stress Disorder - Reaction Index (PTSD-RI) in Zambia.

**Methods:**

The PTSD-RI was adapted through the addition of locally relevant items and validated using local responses to three cross-cultural criterion validity questions. Reliability of the symptoms scale was assessed using Cronbach alpha analyses. Discriminant validity was assessed comparing mean scale scores of cases and non-cases. Concurrent validity was assessed comparing mean scale scores to a traumatic experience index. Sensitivity and specificity analyses were run using receiver operating curves.

**Results:**

Analysis of data from 352 youth attending a clinic specializing in sexual abuse showed that this adapted PTSD-RI demonstrated good reliability, with Cronbach alpha scores greater than .90 on all the evaluated scales. The symptom scales were able to statistically significantly discriminate between locally identified cases and non-cases, and higher symptom scale scores were associated with increased numbers of trauma exposures which is an indication of concurrent validity. Sensitivity and specificity analyses resulted in an adequate area under the curve, indicating that this tool was appropriate for case definition.

**Conclusions:**

This study has shown that validating mental health assessment tools in a low-resource country is feasible, and that by taking the time to adapt a measure to the local context, a useful and valid Zambian version of the PTSD-RI was developed to detect traumatic stress among youth. This valid tool can now be used to appropriately measure treatment effectiveness, and more effectively and efficiently triage youth to appropriate services.

## 2. Background

Sexual violence, particularly discussed in reference to females, is an enormous global health and human-rights problem, and a growing concern in sub-Saharan Africa. According to WHO, about 150 million girls had experienced sexual violence in 2002 [1]. Peer-reviewed research on the sexual abuse of children (CSA) in sub-Saharan Africa is limited and is largely confined to South Africa, yet there is growing evidence of its enormity [2,3].

Sexual violence has devastating short- and long-term mental, reproductive, economic, and physical health consequences [4-6]. With relation to mental health problems, one review of the current literature found that across multiple methodologies, samples and measures, survivors of CSA suffered from the following problems: psychotic symptomatology, depression, PTSD, dissociation, eating disorders, somatization, personality disorders, self-esteem and self-concept impairment, suicidal and self-injurious behavior and substance abuse. In terms of sexuality related issues, problems of sexual dysfunction and engagement in high-risk sexual behaviors (such as unprotected sexual intercourse, sex with multiple partners, early involvement in sexual activity, and prostitution) were reported. Additionally, problems of social impairment, interpersonal problems (including feelings of inadequacy, inferiority, or discomfort when interacting with others), hostility, anger, perpetration of sexual abuse, learning impairment, re-victimization, chronic non- cyclical pelvic pain, and non-epileptic seizures were also identified [7].

One of the most concerning consequences of CSA is the link with HIV transmission through risky sexual behaviors [8-10]. Sub-Saharan Africa is the region most heavily affected by HIV worldwide--accounting for 67% of people living with HIV in 2007 [11]. High prevalence rates are thought to be fueled by early initiation of sex, unprotected sex with non-regular partners, concurrent sexual partnerships, low incidence of condom use among high risk groups and individuals, sexual violence against women, and poverty that forces women and girls to sell sex for food, good grades, small gifts, or money. Zambia is a sub-Saharan country deep in the HIV/AIDS epidemic with an estimated 16% of Zambia adults being HIV+ (women - 18%, men - 13%) [11]. At the time of this study, there were no available studies examining the prevalence of mental health problems in Zambia. A better understanding of sexual violence and its negative sequelae is critical for countries such as Zambia given the relationship between CSA and the HIV/AIDS epidemic.

Although, essential to understanding sexual violence and its consequences, assessing child and caregiver mental health in low-resource countries is a challenging task for researchers and international aid organizations alike [12,13]. Difficulties with mental health assessment include lack of valid instruments, lack of consistent assessment tools for measuring psychological distress, variation in methods for validity testing, and differences in methods of translation [12]. Typically, examining mental health cross culturally involves transporting western assessment tools with no examination of their validity. Although there has been an increased demand for recognizing the effect of culture and context on the understanding and expression of mental health in low-resource countries [14-16], it remains common practice to assume local validity of measures developed in the West. While this remains the case, optimal assessment of local incidence, prevalence, severity and treatment impact requires the use of culturally validated instruments.

In order to address this issue, Bolton and colleagues developed a cross-cultural methodology for assessing local validity of assessment tools. These methods have now been used in multiple studies across several low resource contexts. Bolton [17] explains that criterion validity, or the validity of an instrument in comparison to a "gold standard", is critical for cross-cultural work. However, the lack of mental health professionals in many low resource countries makes using the standard process of a psychiatric diagnosis as the gold standard generally impossible. As an alternative to this 'gold standard' process, Bolton [17] suggests the use of input from the local community to assess criterion validity. The rationale is that if a mental health syndrome occurs in a population and is recognized by the local people then their assessment of the presence/absence of the syndrome could be used as a local criterion. In the work that forms the basis of this approach, Bolton [17] used Key Informants from a community in Rwanda to identify samples of individuals with and without a locally defined syndrome (i.e., agahinda gakabije). The identified individuals were interviewed and asked to self-identify whether or not they themselves thought they suffered from the syndrome. Data from the concordant pairs (yes/yes and no/no) were compared to assess the ability of the syndrome measure to discriminate between those most likely to have the disorder (the yes/yes group) and those most likely not have the disorder (the no/no group). This methodology has since been successfully used in multiple studies with both adults [see [18] for example] and youth [see [19] for example]. This methodology relies on the local population's recognition of problems in the absence of the typical gold standard comparison. We have found this process to provide sufficient local validity to appropriately assess mental health needs as well as evaluate mental health service impact.

This study is part of a larger project within Lusaka, Zambia to implement and evaluate mental health services for youth who have experienced traumatic events and are affected by HIV. In 2004, a qualitative study was conducted to learn about problems of women and children affected by HIV [20]. During this initial study participants were asked what they felt were the problems of women and children in their community and subsequently to describe someone experiencing those problems. The respondents consistently reported high rates of different types of trauma including sexual abuse, physical abuse, and domestic violence, and related local trauma symptomatology. Symptoms resulting from these traumatic experiences included "crying," "thinking too much," "alone and withdrawn," "fearful that it will happen again," "feeling used," "looking confused," "damaged psychologically," "feeling rejected," "shy," "difficulty concentrating," "feeling uneasy and surprised," and "having an unsettled mind--thinking about what has happened". While some of the symptoms were conceptualized as closely resembling the Western classification of PTSD, many were believed to represent different concepts. As a result there was a question as to whether or not Western tools commonly used to measure the trauma-related symptomatology would be applicable or valid within Zambia. This paper presents a study to address this gap, including a process used for incorporating culture-specific information into the adaptation and validation of a trauma-focused mental health assessment tool, the UCLA Child Post-traumatic stress disorder - Reaction Index [21].

## 3. Methods

### 3.1 Setting and Interviewers

This study was conducted within a "One-Stop Centre" at the University Teaching Hospital in Lusaka, Zambia directed by one of the authors (EC). This centre was created to provide medical, legal, and psychosocial evaluation for sexually abused youth, with a particular focus on providing HIV testing and post-exposure HIV prophylaxis for those abused within 72 hours [22]. Four centre staff (3 nurses and 1 social worker) were trained by the first author (LM) to administer the adapted mental health assessment tool (described below) with sexually abused youth and their caregivers. All staff spoke English and several of the local languages (e.g., Nyanja, Bemba, Tonga).

### 3.2 Assessment Tool

Initial development of the measure took place in a meeting with key stakeholders and local experts including the director (EC) and staff of the One-Stop Center, several local medical school students who had expressed interest in mental health issues, a British Psychiatrist who had worked on child sexual abuse issues in Zambia, and a faculty member from the University of Zambia who had also worked with the issue child sexual abuse. With these local stakeholders, the research team reviewed widely used measures of posttraumatic stress disorder (PTSD), depression, anxiety and general externalizing symptoms, together with the results of the prior Zambian qualitative study [20] to select instruments for adaptation and validation. The review specifically focused on determining how closely the characteristics of the problem assessed by the instruments matched those described by the local informants in the preliminary qualitative study as well as determining the extent to which the reviewers thought the full range of mental health problems of HIV-affected sexually abused youth were represented. Although some of the local stakeholders did have expertise in mental health (i.e, the British Psychiatrist and the Zambian clinical psychologist from the University), they both agreed that perhaps they were not intimately familiar with the local terms and perceptions of mental health, and that their Western training and conceptualization may indeed affect this ability. All the stakeholders agreed that it was simply not feasible for either of these high-standing individuals in Zambia to take on the task of interviewing children to represent the "gold standard", and that this model of testing validity would not "fit" into the structure of the One-Stop Center. This paper will focus on an alternative method for adaptation and validation of the measure selected to assess PTSD, the UCLA Child Post-Traumatic Stress Disorder Reaction Index Revision (PTSD-RI) [21,23,24]. While the target population for the One-Stop Centre is children who have experienced sexual abuse, the staff and stakeholders recognized that many of the children will have experienced a wide range of traumatic experiences beyond the abuse, and the PTSD-RI allows for the assessment of multiple traumatic exposure and the accompanying reactions.

The PTSD-RI is a self-report instrument designed to assess trauma exposure and post-traumatic stress experiences and symptoms among children and adolescents. The measure contains three parts, 1) a section on exposures to 12 different traumatic events; 2) a section with 12 questions related to the objective and subjective experiences and memories of the traumatic event; and 3) a section on the frequency of occurrence of 20 specific post-traumatic stress symptoms during the past month. The instrument, one of the most widely used measures of childhood PTSD, has been used internationally in countries such as Armenia [25-30], Turkey [31], Taiwan [32], Bosnia [33,34], Mozambique [35], Kuwait [36], Israel [37,38], Palestine [39] and Lebanon [40]. There have been multiple studies on the psychometric properties of the PTSD-RI showing satisfactory internal consistency [41,42], concurrent validity [43,23,30], and discriminant validity [44]. All of these studies were either done in the West, or in a country where the "gold standard" method of validity testing was possible. To our knowledge the PTSD-RI (or any other mental health assessment tool) has not been validated in Zambia. Since we were unable to use the gold standard method of utilizing clinical interviews to establish case identification, we chose this alternative method for examining validity [17]. For the purpose of this report, we are focusing our validation process on sections one and three of the instrument, the trauma events and symptoms sections.

The process used to adapt the PTSD-RI consisted of working with local collaborators (local authors EC, MI, and several local interviewers from the qualitative study) to review the instrument item-by-item along with the qualitative results, as well as bringing in their local knowledge. For the trauma exposure section, our local collaborators felt every traumatic experience was appropriate. They mentioned that some natural disasters, such as earthquakes, did not happen in Zambia but that a number of children may have moved from other countries where this may have happened. Local collaborators indicated that no additional traumas needed to be added to make it locally relevant. For the symptoms section, the local collaborators identified which signs and symptoms in the PTSD-RI were also identified as problems by the local population in the qualitative study. Where the authors and the local collaborator felt that commonly mentioned signs and symptoms from the qualitative data were not represented in the measure, these were added. For example, "I cry" and "I think too much" were mentioned by multiple Key Informants in the qualitative study, and there were no items on the PTSD-RI that our local collaborators felt expressed the same meaning. Rather than attempt to try to "fit" these signs and symptoms within Western diagnostic boxes, the authors conceptualized these as part of a local presentation of trauma-related problems. This resulted in 18 additional locally relevant signs and symptoms (Table 1) being added. Signs and symptoms in the original PTSD-RI that were not mentioned during the qualitative study were kept in the measure for the validation study as we could not be sure of the reason why they were not mentioned (i.e. were they not relevant, were they very rare, etc.). Keeping them in the measure allowed us to see whether they were still relevant during the validation study. The symptom section of the adapted PTSD-RI thus included 38 total symptom questions.

**Table 1:** Items in the PTSD-RI symptom section

Original Items	Locally Specific Items
1. I watch out for danger or things that I am afraid of.	1. I cry

2. When something reminds me of what happened, I get very upset, afraid or sad.	2. I think too much

3. I have upsetting thoughts, pictures, or sounds of what happened come into my mind when I do not want them to.	3. I have stopped going to school because I think I will be laughed at or teased

4. I feel grouchy, angry or mad.	4. I feel used

5. I have dreams about what happened or other bad dreams.	5. I do not look like myself

6. I feel like I am back at the time when the bad thing happened, living through it again.	6. I am reserved. I cannot open up.

7. I feel like staying by myself and not being with my friends.	7. I am damaged psychologically.

8. I feel alone inside and not close to other people.	8. I feel rejected, like everyone is against me.

9. I try not to talk about, think about, or have feelings about what happened.	9. I feel shy.

10. I have trouble feeling happiness or love.	10. I sleep too much.

11. I have trouble feeling sadness or anger.	11. I do not feel at ease.

12. I feel jumpy or startle easily, like when I hear a loud noise or when something surprises me.	12. I do not feel free.

13. I have trouble going to sleep or I wake up often during the night.	13. I am surprised.

14. I think that some part of what happened is my fault.	14. I am ever quiet

15. I have trouble remembering important parts of what happened.	15. I am unhappy or sad

16. I have trouble concentrating or paying attention.	16. I am nervous.

17. I try to stay away from people, places, or things that make me remember what happened.	17. I have an unsettled mind, no peace of mind.

18. When something reminds me of what happened, I have strong feelings in my body like my heart beats fast, my head aches, or my stomach aches.	18. I run if I see the abuser

19. I think that I will not live a long life.	

20. I am afraid that the bad thing will happen again.	

This process of using local qualitative data to inform the adaptation of a measure has been used in past studies when the locally identified symptoms and problems are similar to recognizable diagnostic categories for which measures exist [17,18]. The presence of additional local symptoms in all of these studies suggest that there may be locally-important mental health symptoms in each context that are not captured in the measures developed for primarily Western populations, reinforcing the importance of adapting measures to each local context.

### 3.3 Translation

The complete PTSD-RI and the cross-cultural criterion validity questions (described below) were translated into Nyanja (the most commonly spoken local language), using a three-step approach: 1) translation-back translation, 2) group translation, and 3) for items that also appeared in the qualitative study, checking the translation results using the local vocabulary. Two multi-lingual translators from the University of Zambia with backgrounds in mental health and an additional multi-lingual translator completed the initial translation-back translation. These translated items were then presented to a group of eight local multi-lingual community members for review symptom by symptom. Each symptom was checked for conceptual understanding as well as the ability for a child and/or community member with limited education to comprehend the language with consensus resulting in the final translation. The determination of whether a child would understand the terms was made by the participants who had children themselves, some of whom were also teachers. Finally, for all key concepts, the words chosen by the translators were compared to the words generated as part of the initial qualitative study. Where there were differences, the term from the qualitative study was used. This occurred infrequently, but when it did occur it was generally around simplifying the grammar and making the wording 'less formal'.

### 3.4 Cross-cultural Criterion Validity Questions

Because there had been interest on the part of the local collaborators to evaluate the validity of the adapted measures, a series of additional questions were added to the end of the caregiver and child assessments precisely for this purpose. These questions specifically asked the child and the caregiver to report the presence or absence of three primary PTSD-related symptoms: Fear, Arousal, and Avoidance. (See Table 2 for a list of the questions). The respondents in the qualitative study did not mention many direct re-experiencing symptoms (e.g., nightmares, flashbacks), which may not be surprising because it has been reported that in children, these symptoms may not be as common [45]. As the formal diagnosis of PTSD was developed based on the experiences and presentation among adults, researchers have documented that the current broad categories (re-experiencing, avoidance, and arousal) may manifest differently in children [45,46]. Re-experiencing in particular tends to manifest itself in wide variation with children and adolescents varying from fear, disruptive behavior, isolation, and/or regressive behaviors [47,48]. In discussion with our local collaborators and referencing the qualitative study, it was decided that the best way to capture the re-experiencing criteria within the Zambian community was to replace reference to specific re-experiencing symptoms with the more general experience of "fear". These three questions formed the basis for our case definition and the cross-cultural validation analysis in this paper. The cross-cultural criterion validity questions were translated using the same methodology described above.

**Table 2:** Cross-cultural Criterion Validity Questions

Child Questions	Caregiver Questions
Do you have fear because of what happened?	Does your child have fear because of what happened?

Do you show signs of avoidance and/or depression?	Does your child show signs of avoidance and/or depression?

Do you show arousal?	Does your child show arousal?

### 3.5 Piloting and Implementation of Instruments

Children and their caregivers came into the One-Stop Centre after already reporting a sexual abuse act to the authorities. As part of the standard process within the One-Stop Centre, these children were offered medical and legal services, and a psychosocial evaluation that provided the basis for referral. The psychosocial evaluation began with both the child and their caregiver being interviewed with a standardized intake form, which included psychosocial assessment measures. For the children, this intake included the adapted version of the PTSD-RI, and the cross-cultural validity criterion questions.

The adapted PTSD-RI was piloted for use in the One-Stop Centre in August 2007. The piloting was done to assure that the translations made sense to the children and caregivers who came to the centre, to allow for live practice for the assessors, and to identify any problems with the implementation process that could be corrected prior to the actual formal assessment initiation in the clinic. All assessments were administered orally by staff trained by one of two authors (LM, KS). The pilot process resulted in no substantial changes to the instrument and thus the complete measure was implemented as part of standard procedures within the One-Stop Centre. The data for this validity study was obtained through a record review of the clinic data. At the time of the analysis, 690 child intakes had been conducted at the centre, with 541 caregivers completing the assessment, which included the caregiver cross-cultural validity questions and 468 children completing the PTSD-RI and child cross-cultural validity questions. For this study, the 352 (75%) children with complete PTSD-RI forms and caregiver data were included in the analysis. Institutional Review Board approval for this analysis was obtained from Johns Hopkins University, Boston University, and the University Teaching Hospital Ethics Board in Zambia.

### 3.6 Data Analysis

Data from two parts of the PTSD-RI were used for this validation analysis, the trauma events and symptom sections. The items from the second part, the objective and subjective experiences were not utilized in this study due to the variation of traumatic events referred to in responding to these (i.e., sexual abuse for some, witnessing violence for others). For the traumatic events data, we looked at the distribution of the 12 individual types of traumas (yes/no response) and created an index by summing the total number of different traumas reportedly experienced by a given child. For the symptom questions, which included the 20 original PTSD-RI symptom questions and the 18 additional locally specific symptoms, we created three summary scores. For each symptom question the child respondents were asked to rate the frequency with which they experienced each symptom in the previous month using a 5-point Likert scale (0 = None of the time, 1 = Little, 2 = Some, 3 = Much, and 4 = Most of the time). The first summary scale was generated by summing the responses for each of the original 20 PTSD-RI items only. The second summary scale was generated by summing the scores of the 18 locally specific symptoms. And a final summary scale was generated by summing the scores of all 38 symptoms. Missing values for individual symptoms were replaced using the mean score of the remaining symptoms from the PTSD-RI and locally specific scales, depending on which scale the missing item was included within. Replacement was only made for respondents with less than 5% missing; this resulted in two cases being dropped from the analysis because of significant missing information.

To assess scale reliability, internal consistency analyses were conducted for each of the scales using Cronbach alpha scores [6]. Both discriminant and concurrent validity were assessed in our analyses. For the discriminant validity analysis, we defined caseness in two different ways. First, we looked individually at the three cross-cultural validity questions. We defined "cases" as those youth for whom the child and caregiver both indicated 'yes' to the presence of a symptom; "non-cases" were those youth for whom both the child and caregiver indicated the specific symptom was not present. Thus we created three sets of "cases" and "non-cases" - one set for each validity question. Second, we defined caseness on the basis of how many of the cross-cultural validity questions the child and caregiver pairs endorsed. Children for whom child/caregiver pairs indicated "no" to all three validity questions were identified as "non-cases", while "cases" were defined as children for whom the pairs indicated 'yes' to at least 2 of the 3 validity questions, regardless of whether they were the same symptoms. This second process was used because we were unsure of the extent to which caregivers and children would be able to correctly ascertain the specific types of problems rather than being able to recognize that there were problems actually present. T-test analyses were used to compare the PTSD-RI symptom scales across the different categories of "cases" and "non-cases". As an indication of discriminant validity, we would expect that "cases" would have, on average, significantly higher scale scores than the "non-cases", indicating the scales' ability to differentiate between the groups.

To assess concurrent validity, we compared the three symptom scale scores with the index of total number of different traumas reportedly experienced by the child. We broke the index up into 4 categories, 0-1 events, 2 events, 3 events, and 4 or more events. As an indication of concurrent validity, we would expect that more reported trauma exposure would correlate with more severe symptoms scores.

To complete the evaluation of the adapted PTSD-RI measure, we conducted sensitivity and specificity analyses using receiver operating characteristic (ROC) curves to test the performance of the symptom scale and suggest appropriate cut-off scores for case-identification.

## 4. Results

Table 3 below presents the basic demographics of the children included in this validity sample (n = 352). Children included were all between the ages of 6 and 15 years, with the sample including primarily girls (98%). More than 80% of the respondents indicated that they were currently attending school. As this sample comes from a clinic specially designated to treat sexually abused youth and make post-exposure prophylaxis available, we also extracted data on the abuse experience. Seventy-two percent of the youth reported that the current abuse was the first time they had been abused, with nearly half of the children (45%) reporting that the abuse occurred within the prior 72 hours. In terms of relationship to the child, the caregivers in our sample were primarily biological mothers (51.5%), biological fathers (13.9%), or "auntie's" (11.3%), with the rest being distributed among adopted parents, grandparents, siblings or other relatives not defined. An "auntie" in Zambia can be anyone who helps to take care of a child including an aunt, neighbor, or friend of the family.

**Table 3:** Child Respondent Demographics and Scale Scores (n = 352)

Age in years, Mean (range)	12.8 (6 - 15)
**Gender N (%)**	

**Male**	7 (2%)

**Female**	345 (98%)

**Currently in School N (%)**	

**Yes**	296 (84.3%)

**No**	55 (15.7%)

Scale Score*, Mean (SD)	

**PTSD-RI scale (20 items)**	16.1 (17.3)

**Locally-specific scale (18 items)**	13.9 (16.5)

**Total Scale (38 items)**	30.0 (32.9)

* The range of possible scores for each of these scales are: PTSD-RI - 0-66 points, Locally-specific scale - 0-72 points, and total scale - 0-135 points.

or the whole sample, the average Carrie run this?e possible range - I think it's more useful to identify the actual range of s

Table 4 presents the prevalence of the different traumatic experiences assessed by the PTSD-RI in this sample. The most endorsed trauma was being sexually touched (63%). Approximately one third of the children reported being shot at, beaten or threatened (38%) or seeing a dead body (29%). None of the children reported being in an earthquake or experiencing war and only 2% reported being in any kind of natural disaster or a bad accident. The mean number of traumas experienced by a child was 2.09 (S.D. 1.36), with the number of reported traumas experienced ranging from 0 - 8.

**Table 4:** Child Endorsements of Traumatic Events

Traumatic Experience	N (%)
1. Being in an earthquake that badly damaged the building you were in	0

2. Being in another kind of disaster, like a fire, tornado, flood or hurricane	7 (2%)

3. Being in a bad accident, like a very serious car accident	6 (2%)

4. Being in a place where a war was going on around you	0

5. Being hit, punched, or kicked very hard at home. DO NOT INCLUDE ordinary fights between brothers and sisters	57 (16%)

6. Seeing a family member being hit, punched, or kicked very hard at home DO NOT INCLUDE ordinary fights between brothers and sisters	60 (17%)

7. Being beaten, shot at, or threatened to be hurt badly in your community	134 (38%)

8. Seeing someone in your community being beaten up, shot at or killed	93 (27%)

9. Seeing a dead body in your community. DO NOT INCLUDE funerals	101 (29%)

10. Having an adult or someone much older touch your private parts when you did not want them to	222 (63%)

11. Hearing about the violent death or serious injury of a loved one	32 (9%)

12. Having painful or scary medical treatment in a hospital or clinic when you were very sick or badly injured	24 (7%)

**Total Number of Reported Traumas, Mean (sd) Range**	2.09 (1.36) (0-8 traumas)

### 4.1 Reliability and Validity of the Symptom Scales

The Cronbach's alpha scores for the 20-item PTSD-RI and the 18-item locally specific scale are .93 and .94, respectively, with no improvement for either scale with the removal of any individual symptom. As an indication of reliability associated with the internal consistency of a measure, Cronbach's alpha scores should be at least 0.7 and ideally greater than 0.8 [49].

For the first analysis of discriminant validity, 195 (55%) of the child-caregiver pairs both reported the presence of "fear" while 31 (9%) both reported the absence of this cross-cultural validity symptom; 34 (10%) both reported the presence of "avoidance" while 163 (46%) both reported the absence of this cross-cultural validity symptom; and 3 (1%) both reported the presence of "arousal" while 276 (78%) both reported the absence of this cross-cultural validity symptom.

For the second analysis of discriminant validity, 29 (8.2%) child-caregiver pairs reported the absence of all three cross-cultural validity symptoms (non-cases) and 38 (10.8%) child-caregiver pairs reported 2 or 3 of the cross-cultural validity symptoms (cases). Table 5 presents the scale score comparison for all the "case" - "non-case" comparisons; all differences were statistically significant, with higher scores for "cases" than "non-cases" across the board.

**Table 5:** Differences in mean scale scores for cases and non-cases for evaluation of discriminant validity of trauma symptoms scales

	Caseness as identified by child and caregiver report		
	**Fear Symptom**		

	**Cases (n = 195)**	**Non-cases (n = 31)**	**p-values**

PTSD-RI symptom scale, mean (SE)	17.3 (1.3)	9.3 (2.7)	0.02

Locally-specific symptom scale, mean (SE)	15.1 (1.2)	8.6 (2.3)	0.04

Total symptom scale, mean (SE)	32.3 (2.4)	17.9 (4.8)	0.02

	**Avoidance Symptom**		

	Cases (n = 34)	Non-cases (n = 163)	

PTSD-RI symptom scale, mean (SE)	26.7 (3.4)	11.0 (1.1)	<0.001

Locally-specific symptom scale, mean (SE)	23.8 (3.2)	9.0 (1.0)	<0.001

Total symptom scale, mean (SE)	50.5 (6.5)	20.0 (2.0)	<0.001

	Validity Question Counts*		

	Cases (N = 38)	Non-cases (N = 29)	

PTSD-RI symptom scale, mean (SE)	25.2 (3.2)	9.4 (2.8)	<0.001

Locally-specific symptom scale, mean (SE)	22.1 (3.0)	9.1 (2.3)	0.002

Total symptom scale, mean (SE)	47.3 (6.1)	18.5 (4.9)	<0.001

* caseness was defined based on the number of cross-cultural criterion validity questions endorsed by the caregiver and child dyads. Endorsing 2 or 3 of the questions defined caseness; endorsing none of the questions defined non-caseness.

For the concurrent validity analysis, Table 6 presents the comparison of symptom scale scores with the traumatic events index categories. For all three symptom scale scores, there was a consistent increase in mean scores across the increasing number of different trauma exposures, with youth reporting the highest category of exposure (4 or more events) having nearly twice the average scale scores compared with youth reporting the lowest category of reported exposure (0 or 1 events).

**Table 6:** Evaluation of concurrent validity: average symptom scale scores by different numbers of reported traumatic events experienced

	Total number of different types of traumatic events*, categorized				
	**0-1** **N = 134**	**2** **N = 86**	**3** **N = 69**	**4 +** **N = 55**	p-value**

**PTSD-RI symptom scale, mean (SD)**	13.13 (15.20)	14.36 (16.82)	17.36 (17.99)	23.13 (20.09)	0.002

**Locally-specific symptom scale, mean (SD)**	11.18 (14.12)	13.20 (17.0)	13.58 (15.64)	20.59 (20.08)	0.005

**Total symptom scale, mean (SD)**	24.31 (28.39)	27.56 (33.02)	30.94 (32.77)	43.72 (39.31)	0.003

*8 records dropped from analysis due to missing trauma event information

** p-value for test of difference in means across groups using ANOVA

**Figure 1:**
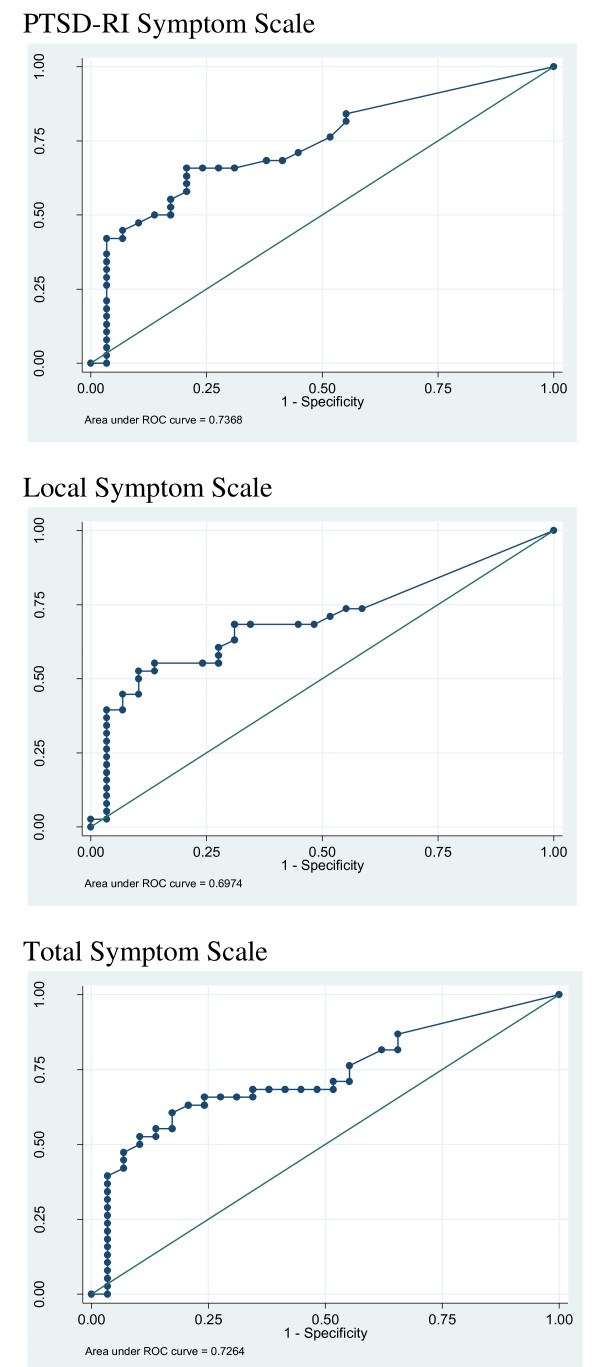
**ROC curves for all three scales**. PTSD-RI Symptom Scale Local Symptom Scale Total Symptom Scale

The results of the sensitivity and specificity analyses are presented in Table 7. For these analyses we used the second method of case definition (i.e. the number of criterion validity questions endorsed). The area under the curve (AUC) ranged from 0.70 to 0.74, depending on the scale being used (Figure 1 presents the ROC curves for all three scales). The 95% confidence interval for all three AUC analyses was greater than .50, indicating that the use of the scale would result in greater than chance results for correctly identifying a true case.

**Table 7:** Test characteristics using receiver operating curves for each of the scales*

	Area under the curve (se), [CI]	Optimal cut-offs	Correctly classified	SENS	SPEC
PTSD-RI symptom scale	0.74 (0.06), [0.62-0.86]	17 points	71.6%	66%	79%

Locally-specific symptom scale	0.70 (0.06), [0.57-0.82]	10 points	68.7%	68%	69%

Total symptom scale	0.73 (0.06), [0.60-0.85]	31 points	70.2%	66%	76%

* caseness was defined based on the number of cross-cultural criterion validity questions endorsed by the caregiver and child dyads. Endorsing 2 or 3 of the questions defined caseness; endorsing none of the questions defined non-caseness.

## 5. Discussion

To our knowledge, this is the first validated trauma-related mental health assessment tool in Zambia. Standard validity testing of psychological assessments is done with a criterion comparison of a structured clinical interview by mental health professionals, and is generally based on strict criteria to a diagnostic category within the DSM or ICD. There are multiple issues with using this procedure across cultures in low and middle income countries [18]. First, there are rarely enough mental health professionals available who are knowledgeable about the local culture and local idioms of mental health symptoms. Second, this procedure is based on a Western diagnostic model that may or may not be applicable across cultures. Third, children and adolescents who have experienced trauma are often difficult to fit into existing diagnostic categories, often presenting with a wide range of symptoms [45]. Therefore, this study did not seek to confirm a diagnosis, but rather to find and validate an instrument that would be useful in identifying local trauma-related symptoms that are seen as problematic within this culture using an alternative and previously used methodology [17-19,50]. Using this methodology, Zambian-specific expressions of trauma obtained from an earlier qualitative study were incorporated into the instrument prior to testing.

The validation method used varied slightly from some past efforts [17,18] in that rather than using a key community member or members to identify those with a disorder, we used the self-report by children and caregivers as to whether the child had problems. This method has been used before by Bolton and colleagues [19], however, research from the West suggests a range of concordance rates between parent and child reports of mental health symptoms in response to a traumatic event [51,52]. Although our study identified a number of discordant caregiver/child dyads, this was expected, and not the focus of the analysis. Our focus was on the most probable "cases" and "non-cases", which we identified by finding the caregiver/child dyads who did agree on the case status. Our assumption is that when there is agreement, then it is closer to the true situation. When there is no agreement, we cannot be sure which respondent to 'believe' and thus their case definition is unclear.

This study showed that this adapted PTSD-RI demonstrated good reliability. Similar to previous studies [21], the Cronbach's alphas demonstrated good internal reliability. The correlations of the local symptoms are comparable to the original 20 items based directly from the DSM criteria suggesting that these are part of the local expression of trauma-related symptoms in children. Although these additional items create a longer measure, our local collaborators expressed interest in retaining them as locally expressed symptoms.

Analysis of the traumatic events data showed many youth reporting more than a single traumatic event. This is similar to other reports of children seeking treatment for sexual abuse [53]. Since the target population was children who were coming into a One-Stop Centre for sexual abuse, a large majority reported sexual abuse (63%). Although it is known objectively, because of their presence in the Centre, that 100% of the population did in fact experience some form of sexual abuse, it is common for children to deny or avoid reporting this type of event(s) or only report one act of abuse out of shame, embarrassment, fear, or to protect someone if more than one event had occurred. By some estimates between 60-80% of CSA victims withhold disclosure suggesting that many children and adolescents endure prolonged victimization and do not receive any therapeutic intervention [54,55]. Studies that examine latency to disclosure report a mean delay from 3-18 years [56]. Disclosure is essential to recovery as literature suggests that simple acts of disclosing past traumatic experiences to others can exert a positive effect on health and well-being [57]. Furthermore, disclosure of CSA in particular is often a prerequisite for access to mental health care services [58,59].

Some adolescents who came to the One-Stop Centre may have denied sexual abuse because they saw the sexual encounter(s) as consensual. There is a phenomenon in Zambia called "sugar daddy" whereby young girls may take up with much older men to receive housing, monies and/or other tangible goods. This 'Sugar Daddy Syndrome' is heavily blamed for not only increased cases of child sexual abuse but also for the spread of HIV [60]. After some time, many adolescents are eventually left or taken back to their families. The parents of such adolescents viewed this situation as sexual abuse because the perpetrators were far older than the adolescents and almost all were over 18 years. These two factors, the shame and/or fear of disclosure and the sense that the experience was not abuse may explain why there were some children (n = 28) reported no traumatic experiences at all, even though we know that they had at least experienced sexual abuse by their presence in the clinic. Denial of trauma is a common problem across all trauma measures [61,62].

In addition to the sexual abuse, there were additional frequently reported traumas including experience of and/or witnessing community violence (38.1%, 26.5% respectively), and seeing a dead body in the community. Although Zambia is a relatively stable country, there is still a degree of violence seen regularly - largely thought to be due to poverty and/or lack of governance (Haworth, unpublished data 2007). A significant number of youth also reported violence in the home, which corroborates with a previous qualitative study where local Zambians reported this to be a significant problem [20]. Overall, our results support the use of a tool asking about different traumatic experiences, even with populations known to have one specific trauma. This also suggests need for assessing traumatic experiences and symptoms among youth in Zambia to obtain appropriate services since the majority experienced multiple traumas (which remains likely to be an under-estimate) [61,62].

This study showed good discriminant validity of the adapted PTSD-RI. Regardless of the case definition used, the average scores on the two subscales (the original 20 items of the PTSD-RI and the added 18 locally-defined items) were statistically significantly higher among the cases compared to non-cases with a large difference. We can look to the standard cut-offs used with the symptom section of the PTSD-RI in Western-based research as an interesting comparison. Research on the PTSD-RI psychometrics suggests a cut-off of 38 having a sensitivity of 0.93 and specificity of 0.87 in detecting PTSD (21). This is slightly higher than our average case scores (using the case definition of number of validity questions endorsed) on the scale using the original 20-items, but likely due to the strict adherence to the DSM criteria and a formal PTSD diagnosis. The non-case mean scores in this study were indicative of those who would not classify as having symptoms based on previous studies using the PTSD-RI [30,42,44].

The significant association of symptom scores across increasing numbers of reported traumas confirmed the expectation that higher exposure would be associated with more severe distress, our assessment of concurrent validity [63-65].

The ROC curve analysis provides evidence that the scales can adequately identify cases and non-cases significantly greater than chance. The area under the curve analyses presents adequate results. In utilizing ROC curves to define cut-off scores, it is necessary to consider a variety of factors such as the types of services provided. For example, for use with community settings where the goal may be to serve all that have any need, a lower cut-off may be chosen to maximize sensitivity. In a clinic-based program which seeks to serve more severe cases, or in a case where limited services are available and the goal is to treat the most severely affected, a higher cut-off score may be selected. Our current analyses do not provide information on what appropriate cut-off scores are for different programs.

Child sexual abuse is increasingly being suggested as a major contributor to the HIV/AIDS epidemic through direct transmission, or indirectly through mental health problems and other high-risk behaviors [3]. HIV/AIDS is one of the most serious public health issues worldwide and sub-Saharan Africa one of the worst affected regions, with significant impact on use of health care services, family and community fabrics, economies, overall quality of life, mortality, and morbidity [66]. It is critical that greater efforts are put towards understanding the nature of sexual violence and its consequences, using validated measures, and promoting treatment for related issues. The staff at the One-Stop Centre found the psychosocial forms helpful in assessing the child's need for services, particularly as their training is mostly medical (personal communication, 2008), and the Centre has continued using these forms. This suggests that incorporating structured mental health assessments into existing medical structures in low-resource countries can be acceptable and feasible.

## 6. Limitations

Self-report instruments were used so responses may have been over- or (more likely) under-reported. Research suggests that traumatized children may significantly under-report trauma symptoms at initial assessments due to avoidance of wanting to talk about a traumatic event [51,62]. In Zambia, youth are likely to be less familiar with disclosing mental health symptoms on a self-report form, which may add to under-reporting. Although the One-Stop Centre staff was repeatedly trained in creating a safe place for the children and administering the forms, which should minimize some under-reporting, it is still a common problem with interview-style assessments [62]. Second, the sample used was homogeneous in respect to all having experienced some form of sexual abuse, and all having already reported the abuse to the authorities. Thus, this is not a community-based sample and does not include children who have not yet disclosed abuse. Despite being homogenous in experience of CSA most of our sample experienced different and multiple types of trauma, suggesting that this measure may be useful for assessment of children with other and multiple types of trauma experiences. Third, of those records reviewed for this study 25% could not be used because we did not have a matching caregiver intake that included the cross-cultural validity questions. Although this is a loss of possible records, given that this was a record review design of forms being newly integrated into a One-Stop Centre in Zambia, we feel that the 75% having matching forms is quite good. Fourth, the cross-cultural criterion validity questions were created through collaboration with local partners examining the qualitative study results, the PTSD diagnostic criteria, and a review of research on how children may present after a traumatic experience. Using cross-cultural validity questions is an alternative way to gauge whether a child was suffering from one of the broad domains of PTSD. Although we feel that these questions captured both the DSM categories, as well as the local presentation, it is known that many children present with wide variation in symptoms that do not always follow the DSM categories [45]. Finally, this study had a specific purpose to analyze whether we could validly discriminate between 'cases' and 'non-cases' in order to have a useful tool for triage in Zambia. If the PTSD-RI measure were to be used for other purposes, further psychometric testing would be warranted.

## 7. Conclusions

This study demonstrates an alternative method of validating child mental health assessment tools in low-resource countries, which may be more feasible than the more typical 'gold standard' methods that utilize clinical diagnosis. Our results suggest that the adapted Zambian version of the PTSD-RI is a valid and reliable mental health assessment tool for detection of traumatic stress symptoms among youth in Zambia.

In low-resource countries, service organizations tend to develop general psychosocial programs and provide them for large populations. These programs commonly assume that all or most youth have similar psychosocial problems and can be helped with the same general program. In truth, the manifestation of child mental health and particularly trauma-related problems is more complex, leaving this practice lacking in cost-effectiveness and treatment appropriateness. There are likely different groups of children who 1) do not present with any symptoms despite being at-risk and/or experiencing multiple trauma and grief experiences, 2) present with mild to moderate symptoms that may be addressed by general psychosocial programs, or 3) present with significant and severe symptoms and need more formalized mental health services. Early identification of mental health problems in youth through locally validated instruments can significantly mitigate numerous problems in adulthood that further tax functioning, well-being, and the greater economy. In summary, taking the time and resources to validate assessment tools is critical to appropriately measure service effectiveness, and more effectively and efficiently triage youth to appropriate services. Policy leaders should begin to require this in all program monitoring and evaluation.

## Declaration of Competing Interests

The authors declare that they have no competing interests.

## Authors' contributions

LKM obtained the funding, conceived and carried out the study, and drafted the manuscript. JB developed and supervised the statistical analysis and assisted with the drafting of the manuscript. EC and MI participated in the coordination of the validation study locally. DT and KS contributed to the design and conceptualization of the study, as well as the ongoing coordination. KS also performed the ongoing data checks. JC contributed to the design of the study and helped draft the manuscript. CL helped perform the statistical analysis and contributed to the manuscript. PB participated in the study design and contributed to the manuscript. All authors have read and approved the final manuscript.
